# Workplace Stress and Well-Being in Nursing: Insights from a Slovenian Cross-Sectional Study

**DOI:** 10.3390/healthcare14060760

**Published:** 2026-03-18

**Authors:** Sebastjan Merlo, Iztok Podbregar

**Affiliations:** 1 Institute of Oncology Ljubljana, 1000 Ljubljana, Slovenia; smerlo@onko-i.si; 2 Faculty of Medicine, University of Ljubljana, 1000 Ljubljana, Slovenia; 3 Angela Boškin Faculty of Health Care, 4270 Jesenice, Slovenia; 4 Faculty of Organizational Sciences, University of Maribor, 4000 Kranj, Slovenia

**Keywords:** workplace stress, nurses, HSE Management Standards, psychosocial work environment, healthcare workforce, Slovenia

## Abstract

**Background:** Work-related stress represents a major challenge for nursing professionals and has significant implications for well-being, job satisfaction, and workforce stability. This study aimed to assess psychosocial working conditions and workplace stress among nurses in Slovenia and to identify organisational and occupational factors associated with stress exposure across different levels of care. **Methods:** A cross-sectional, non-experimental study was conducted using an online self-administered questionnaire. The sample included 736 nurses employed in outpatient settings, hospital wards, and high-intensity care units. Work-related stress was assessed using the Health and Safety Executive (HSE) Work-Related Stress Indicator Tool. Group differences were examined using the Kruskal–Wallis test with Bonferroni-adjusted post hoc comparisons. Associations between HSE dimensions and sociodemographic and work-related variables were analysed using Spearman’s correlation coefficients and multiple linear regression models. **Results:** Statistically significant differences between job positions by level of care were observed for all HSE domains except Demands. Differences in psychosocial working conditions were observed across levels of care, with several domains showing more favourable scores in outpatient and hospital ward settings compared with high-intensity care environments. Regression analyses identified job position by level of care, education level, income, workload indicators, continuous healthcare provision, and job mobility intentions as significant predictors across multiple HSE dimensions. Correlation analyses revealed consistent associations between adverse psychosocial working conditions, increased workload, and indicators of job mobility. **Conclusions:** This study shows that psychosocial working conditions among nurses in Slovenia differ by level of care, with several domains showing more favourable scores in outpatient and hospital ward settings than in high-intensity care environments. Work organisation—especially workload, role clarity, and managerial and peer support—was central to stress, linking adverse conditions to workforce instability and retention risks.

## 1. Introduction

Work-related stress among nursing professionals is a major occupational health concern with important consequences for individual well-being, quality of care, and healthcare system sustainability. Nurses are exposed to high workload, time pressure, shift work, emotional labour, and increasing administrative demands, and sustained exposure to these stressors has been associated with burnout, reduced job satisfaction, impaired quality of life, and increased turnover intention [1,2,3]. Large-scale studies further demonstrate that nurse well-being is closely linked to patient outcomes and workforce stability. Aiken et al. showed that compromised well-being among nurses and physicians is associated with higher turnover, poorer care quality, and increased patient safety risks, indicating that occupational stress represents a systemic organisational challenge rather than solely an individual issue [4]. Similar findings across healthcare systems suggest that work-related stress in nursing is a global phenomenon [5,6]. High stress levels are consistently associated with emotional exhaustion and reduced professional fulfilment, whereas job satisfaction and organisational resources—such as leadership support, role clarity, adequate staffing, and participation in decision-making—appear to mitigate stress and reduce turnover intentions [7,8,9,10,11]. Emotional demands further contribute to occupational stress, particularly in high-intensity settings such as oncology, emergency departments, and intensive care units, where nurses are vulnerable to compassion fatigue and secondary traumatic stress [12,13,14]. Interpersonal conflict and workplace bullying additionally exacerbate stress and burnout, negatively affecting professional quality of life and retention [15]. The Health and Safety Executive (HSE) Management Standards framework provides a structured organisational model for assessing psychosocial risk factors across seven domains (Demands, Control, Managerial Support, Peer Support, Relationships, Role, and Change). These domains capture key organisational conditions that shape the psychosocial work environment in healthcare settings. In nursing practice, they reflect factors such as workload intensity, autonomy in clinical decision-making, leadership support, team collaboration, clarity of professional roles, interpersonal climate, and the management of organisational change. Unfavourable conditions within these domains have been associated with increased stress, burnout, and reduced well-being among healthcare professionals [16,17,18]. Although international evidence is substantial, studies applying standardised organisational stress assessment tools such as the HSE Indicator Tool remain limited in certain contexts, including Slovenia, and comparative analyses across levels of care are scarce. Given the structural differences between outpatient, inpatient, and high-intensity care settings, a better understanding of psychosocial working conditions across these environments is needed to inform targeted organisational interventions. The aim of this study was to examine psychosocial working conditions and work-related stress among nurses across different levels of healthcare provision in Slovenia and to identify organisational and occupational factors associated with variation across the HSE Management Standards domains.

## 2. Materials and Methods

### 2.1. Study Design

This research was conducted as a cross-sectional, non-experimental quantitative study aimed at assessing work-related stress among nursing personnel in Slovenia and identifying organisational and psychosocial factors associated with stress exposure. Data were collected using an online self-administered questionnaire, allowing participants to complete the survey independently and anonymously. The questionnaire was semi-structured and consisted of three sections including sociodemographic information, work-related characteristics, and the validated HSE Work-Related Stress Indicator Tool.

### 2.2. Population and Sampling Strategy

The study population included nurses and nursing associates employed in Slovenian healthcare institutions. Given the size and dispersion of the nursing workforce across different healthcare settings, a convenience sampling approach was applied. Although this sampling method may limit the generalisability of findings, it enabled the inclusion of participants from a wide range of clinical environments. Eligibility criteria were defined as holding a formally recognised nursing qualification (secondary-level nurse, bachelor’s degree nurse, or master’s degree in nursing) and spending at least 70% of working time in direct patient care. Nurses working in outpatient clinics, inpatient hospital wards, emergency departments, intensive care and therapy units, operating rooms, and labour and delivery units were eligible to participate. Participation was voluntary. Participants were categorised according to their primary work setting into outpatient care, inpatient hospital care, and high-intensity care settings (emergency, intensive care, operating and delivery units). These care contexts differ substantially in workload intensity, organisational structure, and exposure to acute clinical situations, which may influence psychosocial working conditions and stress exposure among nursing staff. Nurses working across several units without a clearly defined primary workplace, those on long-term sick leave or maternity leave, and those employed exclusively in managerial roles without direct patient contact were excluded. An a priori power analysis indicated that a minimum of approximately 200 participants per sector would be required to achieve a statistical power of at least 0.80 for planned multivariate analyses at a significance level of α = 0.05.

### 2.3. Instrumentation

The questionnaire consisted of three sections. The first section collected sociodemographic information, including age, gender, education level, years of professional experience, and employment status. Place of residence was included as a contextual variable reflecting potential urban–rural differences in commuting patterns, access to healthcare institutions, and working conditions across Slovenian regions. The second section gathered work-related characteristics, such as clinical unit, shift work, percentage of time spent in direct patient care, and managerial responsibilities. The third section included the previously validated Health and Safety Executive (HSE) Work-Related Stress Indicator Tool [19,20], a validated instrument developed to assess key organisational sources of work-related stress. The HSE tool comprises 35 items, organised into seven dimensions reflecting the Management Standards for work-related stress: Demands (workload, work patterns, and work environment), Control (how much influence individuals have over the way they work), Managerial Support, Peer Support, Relationships (including conflict and unacceptable behaviour), Role (clarity of role and role conflict), Change (how organisational change is managed and communicated). Items are rated on a 5-point Likert-type scale, ranging from 1 (never) to 5 (always). In contrast to many stress measures, higher scores indicate more favourable working conditions and lower exposure to work-related stress. Negatively worded items were reverse-scored in accordance with the HSE scoring guidelines to ensure consistent interpretation across dimensions. Dimension scores were calculated by averaging the relevant items for each scale, with higher mean scores reflecting better psychosocial working conditions. The interpretation of results followed the HSE benchmarking approach, where lower scores indicate areas requiring organisational intervention. Internal consistency reliability was assessed using Cronbach’s alpha coefficient. In the present sample, the overall reliability of the instrument was α = 0.784. Reliability coefficients for individual HSE dimensions were as follows: Demands (α = 0.771), Control (α = 0.788), Managerial Support (α = 0.786), Peer Support (α = 0.774), Relationships (α = 0.780), Role (α = 0.779), and Change (α = 0.784). These values indicate acceptable internal consistency across the domains of the instrument. The questionnaire was translated into Slovenian and subsequently reviewed for linguistic and conceptual equivalence. The translated version was examined by researchers with expertise in occupational health and healthcare management to ensure semantic consistency and contextual appropriateness for Slovenian healthcare settings. A pilot test (*n* = 15) was conducted to assess clarity, comprehensibility, and completion time; no substantive revisions were required following pilot testing.

### 2.4. Data Collection Procedure

Data were collected between March 2025 and May 2025 using the 1KA online survey platform (https://www.1ka.si/). The survey link was disseminated through professional networks and publicly accessible online channels commonly used by nursing professionals. The online questionnaire required responses to the relevant items prior to submission; therefore, the dataset used for statistical analysis contained no missing values for the variables included in the models. Prior to accessing the questionnaire, participants were presented with an information page explaining the study objectives, confidentiality measures, voluntary nature of participation, and data protection procedures. Electronic informed consent was required before proceeding. No personally identifiable data were collected. All responses were anonymised and securely stored. After data collection, the dataset was exported to IBM SPSS Statistics (version 27) for statistical analysis.

### 2.5. Statistical Analysis

Statistical analyses were performed using IBM SPSS Statistics. Descriptive statistics (means, medians, standard deviations, interquartile ranges, frequencies, and percentages) were used to summarise participant characteristics and HSE dimension scores. For selected demographic variables that approximated a normal distribution (e.g., age groups and years of professional experience), one-way analysis of variance (ANOVA) was additionally performed to examine group differences. Normality of continuous variables was assessed using the Kolmogorov–Smirnov test. As several variables did not meet normal distribution assumptions, non-parametric statistical tests were primarily applied. Differences in work-related stress dimensions across clinical settings and professional groups were examined using the Kruskal–Wallis test, followed by Dunn’s post hoc test with Bonferroni correction where appropriate. Associations between HSE dimensions and selected sociodemographic and work-related variables were analysed using Spearman’s rank correlation coefficients. Prior to regression modelling, standard diagnostic procedures were applied. Regression assumptions were evaluated through inspection of residual distributions and residual plots. The selection of predictors was guided by previous research linking sociodemographic characteristics, organisational factors, and employment conditions to occupational stress and psychosocial working conditions in nursing. Predictors included sociodemographic variables (age, education level, income, place of residence), occupational characteristics (job position by level of care, tenure in current position, working hours), and organisational indicators (continuous healthcare provision, compensation mechanisms, and job mobility intentions). Multicollinearity among predictors was assessed using variance inflation factors (VIF), and no problematic collinearity was identified. Model explanatory power was evaluated using R^2^ and adjusted R^2^ values. Categorical predictors were entered into the regression models using dummy coding, with one category serving as the reference group. All predictors were entered simultaneously using the standard (forced entry) method. To identify independent predictors of adverse psychosocial working conditions, multiple linear regression analyses were conducted with individual HSE dimension scores as dependent variables. The regression model was used to assess the relative contribution of each predictor while controlling for the effects of the other variables included in the model. A *p*-value < 0.05 was considered statistically significant.

### 2.6. Ethical Considerations

Ethical approval for the study was granted by the Ethics Committee of the Faculty of Organizational Sciences, University of Maribor (Approval Number: 514-15/2025/2/250-DJ; issued 20 February 2025). All research procedures followed the principles of the Declaration of Helsinki, national legislation, and the General Data Protection Regulation (GDPR).

## 3. Results

A total of 736 nurses were included in the analysis and categorised according to their job position by level of care: outpatient settings (Group 1, *n* = 220), hospital wards (Group 2, *n* = 240), and emergency departments, intensive care and therapy units, as well as operating and delivery units (Group 3, *n* = 276). No statistically significant differences were observed between groups with respect to age (*p* = 0.124) or total work experience (*p* = 0.173). Similarly, no significant between-group differences were found for place of residence (*p* = 0.111) or work schedule (*p* = 0.239), indicating a largely comparable sociodemographic profile across job positions. Additional sociodemographic variables, including gender, education level, and income, were included in subsequent correlation and regression analyses as potential predictors of psychosocial working conditions (Table 1 and Table 2).

Descriptive statistics for all Health and Safety Executive (HSE) Management Standards domains are presented in Table 3 and Table 4. Across the total sample, the highest median scores were observed for *Role* (4.0–4.4) and *Peer Support* (3.5–4.0), while comparatively lower median values were observed for *Demands* (2.88–3.00) and *Relationships* (2.25–2.75). Median scores varied across domains. Hospital wards (Group 2) showed relatively favourable values in several domains, whereas outpatient settings (Group 1) demonstrated higher scores in domains such as Control, Peer Support, Role, and Change. This pattern was evident for *Control*, *Managers’ Support*, *Relationships*, and *Change*. Groups 1 and 3 generally demonstrated lower and, for several domains, comparable median values. The Kruskal–Wallis test (Table 3) identified statistically significant between-group differences for *Control* (*p* < 0.001), *Managers’ Support* (*p* = 0.026), *Peer Support* (*p* < 0.001), *Relationships* (*p* < 0.001), *Role* (*p* < 0.001), and *Change* (*p* < 0.001). No statistically significant between-group differences were observed for *Demands* (*p* = 0.091). Effect size estimates (ε^2^) for the Kruskal–Wallis tests indicated generally small to moderate effects across the domains, with the largest effect observed for the Role domain (ε^2^ = 0.073).

Post hoc analyses with Bonferroni correction revealed distinct patterns across domains. For *Control*, nurses employed in outpatient settings (Group 1) reported significantly higher median scores compared with both Group 2 and Group 3, while no difference was observed between Groups 2 and 3. In contrast, *Managers’ Support* differed significantly only between Groups 2 and 3, with no significant differences involving Group 1. For *Peer Support* and *Relationships*, Group 1 demonstrated significantly higher median scores compared with Groups 2 and 3, whereas no statistically significant differences were observed between Groups 2 and 3. A similar pattern was observed for *Role*, with Group 1 reporting higher median scores than both Groups 2 and 3. For *Change*, Group 1 reported significantly higher median scores compared with Groups 2 and 3, while no significant difference was found between Groups 2 and 3.

Spearman correlation analysis revealed multiple statistically significant associations between sociodemographic, occupational, and organisational variables and the HSE domains (Table 5). Job position by level of care demonstrated significant correlations with all HSE domains except *Demands*, with the strongest association observed for *Role* (ρ = −0.264, *p* < 0.001). Indicators of job mobility showed consistent associations across several domains. Intention to change jobs outside the healthcare system was significantly correlated with *Control*, *Peer Support*, *Relationships*, *Role*, and *Change*. Continuous healthcare provision was significantly correlated with *Control*, *Peer Support*, *Relationships*, *Role*, and *Change*, whereas permanent on-call duty showed no statistically significant correlations with any HSE domain.

Results of the multiple linear regression analyses for individual HSE domains are summarised in Table 6. After adjustment for sociodemographic, occupational, and organisational variables, job position by level of care emerged as a statistically significant predictor for several domains. Job position was independently associated with *Demands* (*p* = 0.046), *Control* (*p* = 0.048), *Peer Support* (*p* = 0.048), *Relationships* (*p* = 0.004), *Role* (*p* < 0.001), and *Change* (*p* = 0.043). No independent association was observed between job position and *Managers’ Support*. Intention to change jobs outside the healthcare system was consistently associated with multiple HSE domains, including *Control* (*p* = 0.029), *Peer Support* (*p* = 0.004), *Relationships* (*p* = 0.015), *Role* (*p* = 0.049), and *Change* (*p* = 0.043). Continuous healthcare provision was independently associated with *Control* (*p* = 0.027) and *Role* (*p* = 0.030). Education level emerged as a significant predictor for *Managers’ Support* (*p* = 0.047) and *Role* (*p* = 0.042). Additionally, income was significantly associated with *Control* (*p* = 0.044), while place of residence was independently associated with *Change* (*p* = 0.047). The explanatory power of the regression models was modest. Adjusted R^2^ values ranged from −0.005 for the Demands domain to 0.072 for the Role domain.

## 4. Discussion

The findings of this study suggest that work-related stress among nurses is shaped primarily by organisational and structural characteristics of work rather than by the specific clinical setting. Uniformly low scores were observed in the Demands domain across outpatient, inpatient, and high-intensity care settings. This pattern indicates that excessive workload and time pressure represent a system-wide stressor in nursing, suggesting that local or unit-level interventions alone are unlikely to be sufficient and also reflect a broader systemic pattern within nursing practice. This supports existing evidence linking high workload and time pressure to burnout, reduced quality of life, and compromised caring behaviours [1,2,3], and is consistent with reports of intensified workload pressures during and after the COVID-19 pandemic [5,6]. From an organisational perspective, the absence of differences in Demands across levels of care is particularly important, as it suggests that workload problems cannot be addressed solely through unit-level interventions. Instead, they point to structural features of healthcare systems, including staffing models, task distribution, and expectations regarding continuous service provision. In the Slovenian context, where workforce shortages and increasing service demands coexist, these findings underline the need for system-level workforce planning and workload regulation strategies rather than isolated local solutions. Similar challenges have been documented internationally, indicating that excessive demands are a common feature of contemporary nursing work across healthcare systems [5,6]. Across the regression models, adjusted R^2^ values ranged from −0.005 (Demands) to 0.072 (Role), indicating modest explained variance. Such levels of explained variance are common in organisational and psychosocial research contexts, where perceived working conditions are influenced by multiple interacting factors. Although job position by level of care emerged as a statistically significant predictor for several HSE domains in the multivariate regression models, this effect should be interpreted within the context of organisational characteristics inherent to different care settings. The pattern of effect sizes observed in the Kruskal–Wallis comparisons was broadly consistent with the regression results. In particular, the Role domain showed both the largest group effect (ε^2^ = 0.073) and the highest explained variance in the regression model (adjusted R^2^ = 0.072), suggesting that differences between healthcare settings play a meaningful role in shaping role clarity and organisational expectations. The findings indicate that observed differences are primarily driven by variations in work organisation, workload structure, and management practices rather than by intrinsic features of specific clinical environments. Differences observed in Control highlight the relevance of work organisation and decision-making structures. Lower perceived control in inpatient and high-intensity settings likely reflects more rigid organisational structures and protocol-driven work processes, highlighting autonomy as a key organisational lever for stress reduction. This aligns with research identifying limited autonomy and constrained decision-making as key contributors to occupational stress and burnout [10,16], particularly in settings characterised by rigid workflows such as intensive care and emergency departments [16,21]. These findings imply that organisational designs allowing greater participation in scheduling, task planning, and clinical decision-making may represent an important protective resource, even in technologically complex environments. The domains of Managerial Support and Peer Support further emphasise the relational dimension of work organisation. Variations across levels of care indicate that leadership practices and team dynamics are not uniform across healthcare settings and may either buffer or exacerbate stress exposure. Supportive leadership has been consistently associated with lower burnout and higher retention [11,17], while collegial support promotes resilience and mitigates emotional strain [17,22]. Conversely, in high-pressure environments, weakened managerial and peer support may amplify emotional exhaustion and compassion-related fatigue [5,13,14,23]. These findings underscore that stress in nursing is not solely a function of workload but is embedded in social and organisational relationships. Results for the Relationships and Role domains point to the importance of clarity and interpersonal climate as components of psychosocial safety at work. Lower scores for workplace relationships in inpatient and high-intensity settings are consistent with evidence linking conflict, intimidation, and bullying to burnout and turnover intention [15,24]. At the same time, the generally favourable Role scores suggest that clear professional responsibilities can function as a stabilising factor in otherwise demanding environments, whereas role ambiguity has been associated with emotional exhaustion and dissatisfaction in complex care contexts [16,25,26]. Together, these findings indicate that organisational interventions targeting communication, conflict management, and role definition may be as important as those addressing workload alone. Challenges identified in the Change domain further illustrate the structural nature of work-related stress. Low scores in this domain, particularly in inpatient and high-intensity care, suggest that organisational change is frequently experienced as poorly communicated and insufficiently participatory. Poorly managed change has been repeatedly identified as a major stressor in healthcare [21,27], and the present findings reinforce the importance of transparent communication, staff involvement, and leadership support during periods of organisational restructuring. This has particular relevance for healthcare systems undergoing continuous reform, where cumulative change processes may erode organisational trust and increase psychosocial risk if not adequately managed. Taken together, these findings suggest that work-related stress in nursing is best understood as an organisational and systemic phenomenon rather than as a problem of individual coping or specific clinical specialties. In Slovenia, where structural pressures on the nursing workforce are increasingly pronounced, the results provide empirical support for organisational strategies focusing on workload regulation, participatory work organisation, leadership development, and the strengthening of team-based support. At the same time, the observed pattern corresponds closely with international evidence, indicating that the mechanisms linking organisational conditions to nurse stress are likely to operate similarly across healthcare systems. For healthcare organisations, these results highlight that addressing work-related stress is not only a matter of employee well-being but also of organisational performance and sustainability. High stress levels have implications for staff retention, quality of care, and patient safety [1,2,3,4], making psychosocial risk management a strategic rather than peripheral concern. Organisational interventions aimed at improving working conditions, clarifying roles, and strengthening leadership and peer support may therefore contribute not only to healthier work environments but also to more resilient healthcare organisations. The relatively modest regression coefficients observed across the models reflect the complex and multifactorial nature of psychosocial working conditions in healthcare environments, where organisational, occupational, and contextual factors jointly contribute to perceived work-related stress. Strengths of this study include the use of a validated and internationally recognised instrument—the HSE Work-Related Stress Indicator Tool—and the inclusion of nurses across multiple levels of healthcare provision. The combination of non-parametric group comparisons, correlation analyses, and multivariate regression modelling enabled a comprehensive assessment of psychosocial working conditions and their associations with work-related stress. Several limitations should be acknowledged. The cross-sectional design precludes causal inference, convenience sampling may limit generalisability, and reliance on self-reported data may have introduced response or social desirability bias. As participation was voluntary and conducted online, some degree of selection bias cannot be excluded. In addition, certain organisational characteristics not captured in the present dataset—such as institutional size, unit-level staffing ratios, or local managerial practices—may also influence psychosocial working conditions and should be considered in future research. From a practical perspective, the findings support organisational-level interventions targeting workload demands, professional autonomy, managerial and peer support, and communication during organisational change. Future research should employ longitudinal and mixed-methods designs to clarify causal pathways and to evaluate how specific organisational interventions and leadership practices influence psychosocial working conditions across healthcare systems.

## 5. Conclusions

This study provides a comprehensive overview of work-related stress and psychosocial working conditions among nurses across different levels of healthcare in Slovenia. The results demonstrate that workplace stress is not uniformly distributed across clinical settings and is strongly influenced by organisational and occupational factors rather than individual characteristics alone. Differences in psychosocial working conditions were observed across levels of care, with several domains showing more favourable scores in outpatient and hospital ward settings compared with high-intensity care environments. Across HSE dimensions, workload-related indicators, continuous healthcare provision, role clarity, and support structures emerged as key factors associated with perceived stress. In addition, indicators of job mobility were consistently linked to less favourable psychosocial working conditions, underscoring the potential relationship between workplace stress and workforce instability. Nurses with intentions to leave the healthcare system experienced less favourable psychosocial conditions, suggesting a close link between workplace stress and workforce instability. The findings indicate that psychosocial working conditions among nurses are shaped primarily by organisational and occupational factors, including workload, work organisation, and support structures. Although job position by level of care remained a statistically significant predictor for several HSE domains, these differences appear to reflect underlying organisational arrangements rather than inherent characteristics of specific clinical settings. This underscores the importance of system-level and organisational interventions to reduce work-related stress across healthcare services. Future research should build on these findings using longitudinal designs to explore causal relationships and to evaluate the effectiveness of targeted stress-reduction interventions in nursing practice.

## Figures and Tables

**Table 1:** Sociodemographic characteristics of the sample by job position and level of care (continuous variables).

Sociodemographic Variables (Linear)	Job Position by Level of Care	Participants No. (N = 736)	Mean (± Std. Dev.)	*p* Value
Age (Years)	1	220	36.90 (± 5440)	0.124
	2	240	37.71 (± 6140)	
	3	276	37.36 (± 5870)	
Total work experience	1	220	14.81 (± 6910)	0.173
	2	240	15.37 (± 6430)	
	3	276	15.48 (± 6490)	

**Table 2:** Sociodemographic characteristics of the sample by job position and level of care (categorical variables).

Sociodemographic Variables (Categorical)		Job Position by Level of Care—N (%)			*p* Value
		1	2	3	
Place of residence	1	121 (55.0%)	112 (56.7%)	160 (58.0%)	0.111
	2	99 (45.0%)	128 (43.3%)	116 (42.0%)	
Work schedule	1	44 (20.0%)	64 (26.7%)	64 (23.2%)	0.239
	2	176 (80.0%)	176 (73.3%)	212 (76.8%)	

**Table 3:** Comparison of Health and Safety Executive (HSE) Management Standards domains across job positions and levels of care.

HSE Category	Group * (Job Position by Level of Care)	Participants (*n*)	Median	*p*-Value **
Demands	1	220	2875 (2125–3500)	0.091
	2	240	3000 (2625–3250)	
	3	276	3000 (2500–3375)	
Control	1	220	3333 (2500–3833)	<0.001
	2	240	3000 (2500–3500)	
	3	276	3000 (2500–3500)	
Managers support	1	220	3000 (2650–3600)	0.026
	2	240	3200 (2600–4000)	
	3	276	3000 (2200–3600)	
Peer support	1	220	4000 (3312–4500)	<0.001
	2	240	3500 (3000–4000)	
	3	276	3750 (3000–4000)	
Relationships	1	220	2250 (1750–3000)	<0.001
	2	240	2500 (2250–3000)	
	3	276	2750 (2250–3250)	
Role	1	220	4400 (4000–4600)	<0.001
	2	240	4000 (3800–4200)	
	3	276	4000 (3600–4200)	
Change	1	220	3333 (2666–3666)	<0.001
	2	240	3000 (2333–3666)	
	3	276	2667 (2000–3666)	

* 1—outpatient settings, 2—hospital wards, 3—emergency departments, intensive care and therapy units, operating and delivery units; ** Kruskal–Wallis test.

**Table 4:** Post hoc pairwise comparisons of Health and Safety Executive (HSE) Management Standards domains between job positions and levels of care.

HSE Category	Intergroup Comparison (Job Position by Level of Care)	*p*-Value **	
		Sig.	Adj. Sig. ***
Demands	1–2	0.042	0.125
	1–3	0.077	0.230
	2–3	0.732	1.000
Control	1–2	0.001	0.004
	1–3	0.002	0.005
	2–3	0.851	1.000
Managers support	1–2	0.618	1.000
	1–3	0.049	0.146
	2–3	0.011	0.033
Peer support	1–2	<0.001	<0.001
	1–3	<0.001	<0.001
	2–3	0.911	1.000
Relationships	1–2	<0.001	<0.001
	1–3	<0.001	<0.001
	2–3	0.351	1.000
Role	1–2	<0.001	<0.001
	1–3	<0.001	<0.001
	2–3	0.037	0.112
Change	1–2	0.004	0.013
	1–3	<0.001	<0.001
	2–3	0.309	0.928

** Pairwise comparisons; *** Significance values adjusted by the Bonferroni correction for multiple comparisons.

**Table 5:** Spearman correlation coefficients between sociodemographic, occupational, and organisational variables and Health and Safety Executive (HSE) Management Standards domains.

HSE—Spearman’s Rho		Demands	Control	Managers Support	Peer Support	Relationships	Role	Change
Age	Corr. Coef.	−0.019	0.013	0.047	0.031	−0.010	−0.011	0.024
	*p*-value	0.602	0.720	0.201	0.393	0.767	0.763	0.510
Education level	Corr. Coef.	−0.022	−0.018	−0.096	−0.048	0.068	−0.004	−0.056
	*p*-value	0.558	0.627	0.009	0.187	0.047	0.912	0.128
Housing status	Corr. Coef.	0.017	0.009	−0.054	−0.033	0.019	0.004	−0.024
	*p*-value	0.632	0.797	0.143	0.366	0.589	0.899	0.511
Income	Corr. Coef.	0.034	−0.061	−0.085	−0.099	0.122	−0.154	−0.139
	*p*-value	0.355	0.095	0.022	0.007	0.001	0.000	0.000
Place of residence	Corr. Coef.	0.002	−0.062	−0.015	0.013	−0.030	0.019	−0.055
	*p*-value	0.946	0.043	0.670	0.727	0.420	0.593	0.136
Total work experience	Corr. Coef.	−0.013	0.008	0.054	0.034	−0.014	−0.021	0.035
	*p*-value	0.725	0.823	0.141	0.349	0.685	0.562	0.339
Tenure in current position	Corr. Coef.	0.034	−0.052	0.013	−0.013	0.051	−0.079	−0.022
	*p*-value	0.354	0.155	0.716	0.719	0.169	0.033	0.550
Job position by level of care	Corr. Coef.	0.060	−0.109	−0.079	−0.147	0.177	−0.264	−0.141
	*p*-value	0.102	0.003	0.032	0.000	0.000	0.000	0.000
Number of working hours	Corr. Coef.	0.028	−0.050	−0.050	−0.077	0.086	−0.074	−0.056
	*p*-value	0.443	0.170	0.167	0.036	0.019	0.046	0.130
Clearly defined job tasks	Corr. Coef.	−0.001	0.012	−0.004	0.022	0.018	0.023	0.050
	*p*-value	0.965	0.739	0.917	0.549	0.629	0.526	0.167
Staffing levels according to standards	Corr. Coef.	0.009	0.034	0.026	0.012	0.016	0.016	0.056
	*p*-value	0.801	0.353	0.489	0.747	0.648	0.669	0.129
Employer	Corr. Coef.	−0.003	0.073	−0.034	0.073	−0.044	0.105	−0.014
	*p*-value	0.914	0.047	0.343	0.049	0.232	0.004	0.699
Financial Performance bonus	Corr. Coef.	0.011	0.005	−0.027	0.001	−0.030	0.080	−0.037
	*p*-value	0.746	0.877	0.472	0.971	0.415	0.029	0.313
Compensation for increased workload	Corr. Coef.	0.007	0.010	−0.045	−0.003	−0.021	0.061	−0.030
	*p*-value	0.850	0.790	0.224	0.935	0.552	0.048	0.415
Overtime work compensation	Corr. Coef.	−0.022	0.028	0.014	0.025	−0.012	0.005	0.011
	*p*-value	0.551	0.456	0.706	0.496	0.745	0.883	0.775
Use of meal break	Corr. Coef.	0.020	−0.012	−0.009	−0.009	0.007	−0.008	0.025
	*p*-value	0.585	0.741	0.813	0.806	0.842	0.830	0.497
Intention for Job change within the healthcare system	Corr. Coef.	0.048	−0.021	−0.031	−0.065	0.055	−0.142	−0.094
	*p*-value	0.188	0.563	0.399	0.045	0.139	0.000	0.010
Intention for Job change outside the healthcare system	Corr. Coef.	0.029	−0.063	−0.045	−0.087	0.075	−0.093	−0.084
	*p*-value	0.424	0.046	0.222	0.019	0.042	0.011	0.022
Utilisation of annual leave	Corr. Coef.	−0.019	0.010	0.043	0.051	0.007	0.036	0.040
	*p*-value	0.603	0.767	0.241	0.164	0.837	0.327	0.269
Work schedule	Corr. Coef.	−0.003	−0.001	−0.006	−0.040	0.008	−0.001	−0.006
	*p*-value	0.930	0.962	0.870	0.275	0.826	0.969	0.861
Continuous healthcare provision	Corr. Coef.	−0.052	0.120	0.032	0.118	−0.130	0.177	0.069
	*p*-value	0.162	0.001	0.383	0.001	0.000	0.000	0.042
Permanent on-call duty	Corr. Coef.	0.032	−0.005	−0.007	0.014	0.005	0.047	−0.009
	*p*-value	0.387	0.890	0.844	0.704	0.887	0.199	0.802

**Table 6:** Multiple linear regression analysis of statistically significant sociodemographic, occupational, and organisational predictors of Health and Safety Executive (HSE) Management Standards domains.

HSE Category	Unstandardised Coefficients B	Standardised Coefficients Beta	95% Confidence Interval for B	*p*-Value
DEMANDS				
Job position by level of care	0.081	0.100	(0.001–0.161)	0.046
CONTROL				
Income	0.129	0.105	(−0.017–0.275)	0.044
Intention for Job change outside the healthcare system	−0.152	−0.094	(−0.288–−0.016)	0.029
Continuous healthcare provision	0.191	0.101	(0.022–0.360)	0.027
MANAGERS SUPPORT				
Education level	−0.131	−0.083	(−0.271–0.009)	0.047
PEER SUPPORT				
Tenure in current position	−0.012	−0.107	(−0.025–−0.001)	0.049
Job position by level of care	−0.104	−0.097	(−0.209–0.001)	0.048
Intention for Job change outside the healthcare system	−0.220	−0.124	(−0.369–−0.070)	0.004
RELATIONSHIPS				
Job position by level of care	0.121	0.143	(0.039–0.203)	0.004
Intention for Job change outside the healthcare system	0.145	0.105	(0.028–0.262)	0.015
ROLE				
Education level	0.073	0.074	(−0.012–0.158)	0.042
Job position by level of care	−0.145	−0.209	(−0.210–−0.079)	<0.001
Intention for Job change outside the healthcare system	−0.090	−0.079	(−0.184–0.003)	0.049
Continuous healthcare provision	0.129	0.096	(0.012–0.246)	0.030
CHANGE				
Place of residence	−0.165	−0.087	(−0.336–0.005)	0.047
Job position by level of care	−0.116	−0.100	(−0.230–−0.003)	0.043
Intention for Job change outside the healthcare system	−0.167	−0.087	(−0.329–−0.005)	0.043

## Data Availability

The data presented in this study are available on request from the corresponding author. The data are not publicly available due to privacy and ethical restrictions.
